# Cell-free methods to produce structurally intact mammalian membrane proteins

**DOI:** 10.1038/srep30442

**Published:** 2016-07-28

**Authors:** Takehiro Shinoda, Naoko Shinya, Kaori Ito, Yoshiko Ishizuka-Katsura, Noboru Ohsawa, Takaho Terada, Kunio Hirata, Yoshiaki Kawano, Masaki Yamamoto, Taisuke Tomita, Yohei Ishibashi, Yoshio Hirabayashi, Tomomi Kimura-Someya, Mikako Shirouzu, Shigeyuki Yokoyama

**Affiliations:** 1 RIKEN Systems and Structural Biology Center, Yokohama 230-0045, Japan; 2 Division of Structural and Synthetic Biology, RIKEN Center for Life Science Technologies, Yokohama 230-0045, Japan; 3 RIKEN Structural Biology Laboratory, Yokohama 230-0045, Japan; 4 RIKEN SPring-8 Center, 1-1-1, Kouto, Sayo-cho, Sayo-gun, Hyogo 679-5148, Japan; 5 Department of Neuropathology and Neuroscience, Graduate School of Pharmaceutical Sciences, The University of Tokyo, Bunkyo-ku, Tokyo 113-0033, Japan; 6 Laboratory for Molecular Membrane Neuroscience, RIKEN Brain Science Institute, Wako, Saitama 351-0198, Japan

## Abstract

The crystal structures of four membrane proteins, from bacteria or a unicellular alga, have been solved with samples produced by cell-free protein synthesis. In this study, for mammalian membrane protein production, we established the precipitating and soluble membrane fragment methods: membrane proteins are synthesized with the *Escherichia coli* cell-free system in the presence of large and small membrane fragments, respectively, and are simultaneously integrated into the lipid environments. We applied the precipitating membrane fragment method to produce various mammalian membrane proteins, including human claudins, glucosylceramide synthase, and the γ-secretase subunits. These proteins were produced at levels of about 0.1–1.0 mg per ml cell-free reaction under the initial conditions, and were obtained as precipitates by ultracentrifugation. Larger amounts of membrane proteins were produced by the soluble membrane fragment method, collected in the ultracentrifugation supernatants, and purified directly by column chromatography. For several proteins, the conditions of the membrane fragment methods were further optimized, such as by the addition of specific lipids/detergents. The functional and structural integrities of the purified proteins were confirmed by analyses of their ligand binding activities, size-exclusion chromatography profiles, and/or thermal stabilities. We successfully obtained high-quality crystals of the complex of human claudin-4 with an enterotoxin.

Membrane proteins, including receptors, channels, and transporters, perform a variety of essential cell functions. Moreover, many drugs in clinical use target membrane proteins. X-ray crystallographic structure studies of such membrane proteins are important to understand their functional mechanisms and to promote drug development. However, the crystal structure determination for membrane proteins is much more difficult than that for globular proteins. In particular, human membrane proteins are difficult X-ray crystallography targets^1^,^2^. For crystallization, human membrane proteins are usually expressed in *Pichia pastoris*, insect, or mammalian cells, rather than in *Escherichia coli*^3^. Unfortunately, the overproduction of membrane proteins in the host cells tends to result in cytotoxicity and poor expression. Membrane proteins may be localized in detergent resistant membrane domains, such as lipid rafts, which are challenging to solubilize. These difficulties are serious hindrances to structural biology analyses.

Fortunately, the cell-free protein synthesis methods have been remarkably improved^4^. For structural biology, the cell-free method with *E. coli* cell extracts stands unrivaled, as it has successfully been used to determine numerous solution and crystal structures of soluble human proteins^5^. Furthermore, the *E. coli* cell-free method is expected to become the standard method for membrane protein preparation toward functional analyses and structural biology^6^,^7^,^8^,^9^,^10^,^11^,^12^,^13^,^14^. Many of the difficulties associated with the cell-based recombinant expression methods are overcome by the use of the cell-free method. For instance, there is no cytotoxicity in the cell-free systems. The expressed proteins are not integrated into detergent resistant membrane domains, and the level of expression is not limited by the capacities of the host cells. The cell-free synthesized membrane proteins can be solubilized with mild detergents, whereas the membrane proteins produced by the cell-based expression often require harsh detergents for liberation from the cell membrane fractions. Furthermore, it is also possible to directly integrate the produced membrane proteins into lipid bilayers^9^. Therefore, a large amount of a highly pure and homogenous sample can be prepared in a cost-effective manner by the cell-free method^3^.

Nevertheless, only four structures of cell-free synthesized membrane proteins have been reported: *E. coli* EmrE^15^, *E. coli* DgkA^16^ and *Acetabularia* rhodopsin (AR) I and II from a unicellular alga^17^,^18^. The cell-free systems for these analyses were derived from *E. coli* cells. In the present study, we aimed at taking full advantage of the *E. coli* cell-free synthesis method for the crystallography of human membrane proteins. We established two complementary cell-free methods, described below, for the production of membrane proteins in lipid environments, and the structural and functional characterization of the products. Thereby, we succeeded in the cell-free expression of 19 mammalian membrane proteins (mostly human), improved the conditions for cell-free protein production, and efficiently prepared purified membrane protein samples with excellent quality for X-ray crystallography.

## Results

### Methods for cell-free membrane protein production for X-ray crystallography

We previously developed an *E. coli* cell-free synthesis method to produce membrane proteins in the presence of detergent and lipid^9^, and successfully applied it to the crystallographic structural determination of the AR I and II proteins^17^,^18^. In this cell-free method, the synthesized membrane proteins are simultaneously inserted into large membrane fragments, which may fuse to form liposomes if the detergent concentration is decreased. The cell-free products are collected in the precipitates by ultracentrifugation at 100,000 *g*. This method is designated hereafter as the precipitating membrane fragment (P-MF) method. The yield of functional membrane protein by the P-MF method is superior to that by the simple addition of preformed liposomes to the cell-free reaction^9^.

In addition, in the present study, we utilized the soluble membrane fragment (S-MF) method, by which the membrane protein is synthesized by the *E. coli* cell-free system and simultaneously integrated into small membrane fragments, and then is collected in the soluble fraction by ultracentrifugation. In the S-MF method, lipid/detergent mixed micelles are included in the cell-free protein synthesis solution at a higher ratio of detergent to lipid than that in the P-MF method. One type of S-MF is the “bicelle”^12^, but we have found better S-MFs than the bicelle with respect to cell-free membrane protein production by the S-MF method. The small membrane fragments used in the S-MF method rarely fuse with each other and should continue to integrate membrane protein molecules simultaneously with their synthesis, because the initial concentration of the detergent is maintained in the reaction solution. Therefore, better membrane protein insertion occurs with the small membrane fragments, as compared to pre-formed liposomes^7^ and nanodiscs^8^.

**Figure 1:**
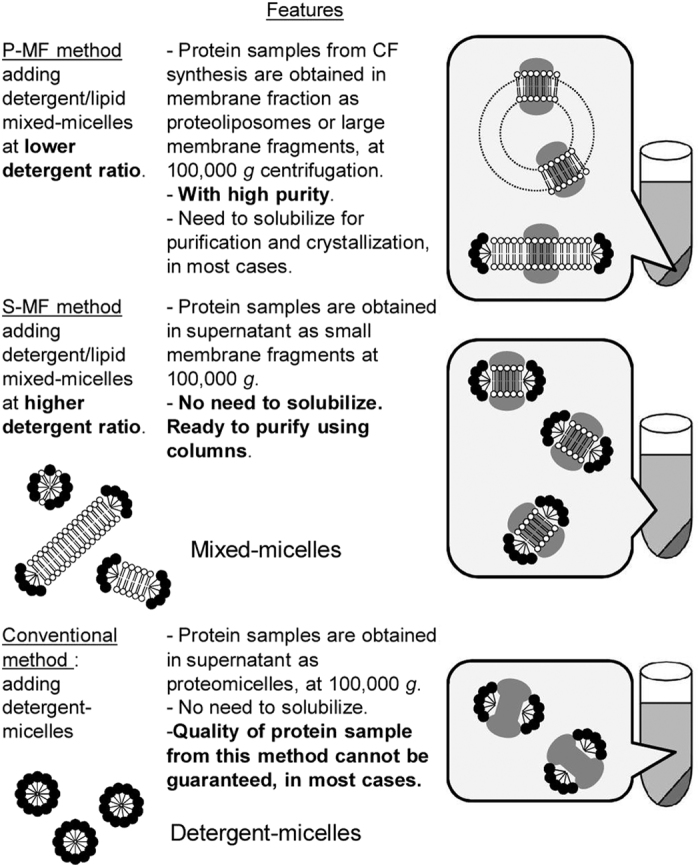
Conceptual diagrams of the *Escherichia coli* cell-free membrane protein production methods. The P-MF method (top), the S-MF method (middle), and the conventional method (bottom).

The characteristics of these two methods are briefly summarized, in comparison with the conventional cell-free synthesis method using detergents, in Fig. 1.

### Combined use of the P-MF and S-MF methods

The P-MF and S-MF methods may be used in combination toward the optimal performance of mammalian membrane protein production. The P-MF method should be used first for testing membrane protein synthesis. Some membrane proteins only correctly exhibit their functions, such as enzymatic activities, when they are inserted into a lipid bilayer, an environment with lateral pressure. The conditions may be improved by the addition of specific mammalian lipids in the P-MF method, with their functions as indicators of structural integrity. However, after optimization of the lipid conditions, the added lipids may have properties related to detergent resistant membrane domain formation, and the membrane proteins may be difficult to solubilize with mild detergents.

The S-MF method should be used in the next step for the production of highly purified uniform membrane proteins with structural integrity in robust yields. The membrane proteins produced by the S-MF method are harvested as ultracentrifugation supernatants, and can readily be purified by column chromatography with no further detergent solubilization step, even after the optimization of the lipid conditions. Therefore, the final yields of the structurally intact membrane proteins produced by the S-MF method are higher than those generated by the P-MF method.

### Cell-free production trials of mammalian membrane proteins under the initial conditions by the P-MF method

**Figure 2:**
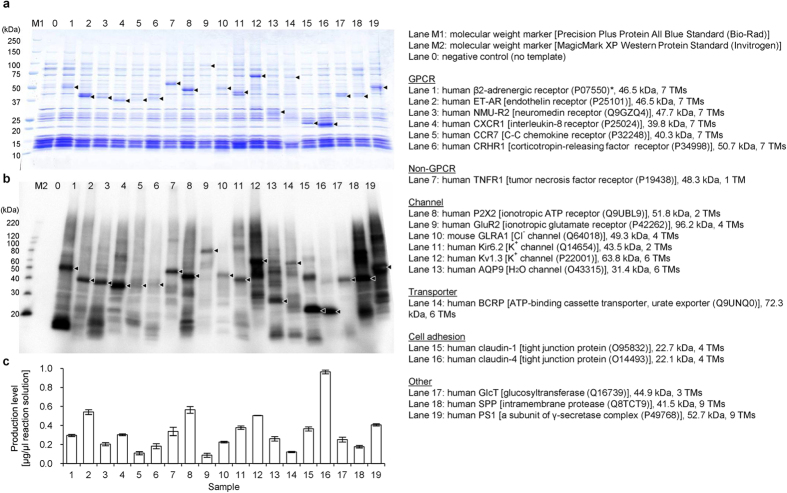
Production of mammalian membrane proteins by the P-MF method under the initial conditions. Cell-free-produced membrane proteins in the membrane fractions were detected by SDS-PAGE and Coomassie Brilliant Blue-staining (**a**), and by western blotting with HisProbe-HRP (**b**). (**c**) Densitometry analysis of the bands of the cell-free-produced membrane proteins in the SDS-PAGE gel (**a**) with means ± S.D. (error bars); *n* = 3. For each lane, the analyzed membrane protein is indicated on the right side of the figure. TM helices, transmembrane helices; GPCR, G-protein-coupled receptor; ET-AR, endothelin-A receptor; NMU-R2, neuromedin-U receptor 2; CXCR1, C-X-C chemokine receptor type 1; P2X2, P2X purinoceptor 2; Glu-R2, α-amino-3-hydroxy-5-methyl-isoxazolepropionic acid-selective glutamate receptor 2; GLRA1, glycine receptor subunit α-1; Kir6.2, ATP-sensitive inward rectifier potassium channel 11; Kv1.3, potassium voltage-gated channel subfamily A member 3; AQP9, aquaporin-9; BCRP, breast cancer resistance protein; GlcT, glucosylceramide synthase; SPP, signal peptide peptidase; PS1, presenilin-1. *Swiss-Prot accession codes of membrane proteins are shown in parentheses.

First, mammalian membrane protein production was tested by the cell-free trial under the initial conditions, utilizing digitonin as the detergent and L-α-phosphatidylcholine from egg yolk (egg PC) as the lipid. Consequently, we successfully produced a set of nineteen mammalian α-helical membrane proteins with fewer than ten transmembrane helices, as follows. If present, the signal sequences of the membrane proteins were omitted. An N-terminal fusion sequence including a modified natural poly-histidine (N11, MKDHLIHNHHKHEHAHAEH) affinity tag (see Methods) was used for all of the membrane proteins, in order to standardize the rate of translation initiation. After the cell-free production reaction, the membrane fraction was collected and analyzed by SDS-PAGE. The nineteen membrane proteins were produced by the P-MF method, under the standard conditions without any optimization (Fig. 2a–c). Each of the cell-free products was detected as the main band by Coomassie Brilliant Blue staining (Fig. 2a). In the western blotting analysis, other bands due to aggregated and fragmented products derived from the cell-free synthesized membrane proteins were detected, in addition to the main bands representing the desired products (Fig. 2b). Generally, however, the proper products were much more abundant than the aggregated/fragmented products (Fig. 2a,b). The production levels actually amount to 0.1–1.0 mg (about 0.3 mg on average) per ml cell-free reaction (Fig. 2c). These standard conditions are also applicable to membrane proteins with more than ten transmembrane helices, because mammalian membrane proteins with twelve or fourteen transmembrane helices were successfully produced by the P-MF method (to be published elsewhere). In the following sections, we describe our results for human glucosylceramide synthase (GlcT), γ-secretase subunits, and claudins.

### Human glucosylceramide synthase (GlcT)

**Figure 3:**
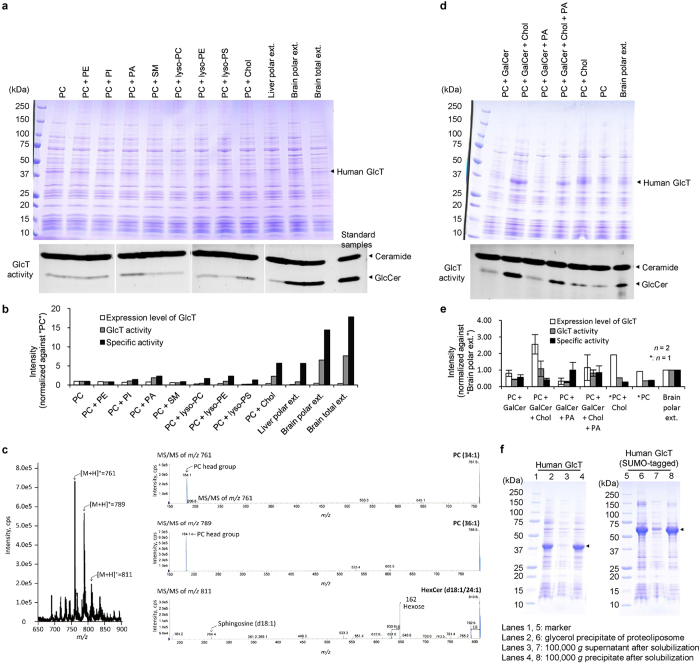
Improvement of the expression level and the specific activity of human GlcT by optimization of the lipid in the cell-free production conditions. (**a**) Human GlcT was expressed by the P-MF method in the presence of various lipids. Under the conditions with the multiple lipids mixture, liposomes containing 95% (w/w) PC and 5% (w/w) added lipid were used for the cell-free production. For example, “PC + PE” means the mixture of 95% (w/w) PC and 5% (w/w) PE. Human GlcT in the membrane fractions was detected by SDS-PAGE with Coomassie Brilliant Blue staining, and the enzymatic activity was assayed by the NBD-labeled ceramide method. (**b**) Densitometry analysis of the SDS-PAGE bands of human GlcT and the TLC spots of GlcCer in (**a**). The densities of the bands and spots were measured with Image J (http://imagej.nih.gov/ij/), and were normalized against “PC”. The specific activities were calculated by dividing the intensities of the TLC spots by those of the SDS-PAGE bands; *n* = 1. (**c**) Specifically bound lipids from the purified human GlcT were detected and identified by the MS/MS method. (**d**) Human GlcT was produced by the P-MF method under the conditions using the effective lipids from (**a**,**c**), with 5% (w/w) additive lipids mixed with PC, respectively. (**e**) Densitometry analysis of the SDS-PAGE bands of human GlcT and the TLC spots of GlcCer in (**d**), performed as in (**b**). The densities of the bands and spots were normalized against “Brain polar ext.”, with means ± S.D. (error bars); *n* = 2. *“PC” and “PC + Chol”; *n* = 1. (**f**) Solubilization of the human GlcT and the SUMO-tagged human GlcT proteoliposomes produced by the P-MF method under the optimized conditions. PE, phosphatidylethanolamine; PI, phosphatidylinositol; PA, phosphatidic acid; SM, sphingomyelin; lyso-PC, lysophosphatidylcholine; lyso-PE, lysophosphatidylethanolamine; lyso-PS, lysophosphatidylserine; Chol, cholesterol; GalCer, galactosylceramide; and GlcCer, glucosylceramide. Experiments were performed either once (**a**–**c**) or twice (**d**–**f**). All gel images in this figure were processed by cropping.

GlcT is a putative three-transmembrane protein that functions as the first enzyme in glycolipid biosynthesis^19^. The yields of GlcT in various cell lines and wheat germ cell-free protein synthesis have been disappointingly low. In contrast, in the *E. coli* cell-free production trial under the standard conditions, GlcT was detectably produced by the P-MF method (Fig. 2a–c, lane 17). The quantity and quality of the cell-free products in large membrane fragments can be analyzed without the preparation of solubilized samples; *e*.*g*., by analyses of the production level and the enzymatic activity, and improved by modifying the conditions. In this context, some membrane proteins require specific lipids for their structural stability and proper function^20^,^21^. Therefore, using the P-MF method, various lipids were tested for their effects on the GlcT production level and enzymatic activity^22^ (Fig. 3a).

As compared with egg PC alone under the standard conditions (Fig. 3a, lane “PC”), the total and polar lipid extracts from porcine brain remarkably increased the specific activity of GlcT by about 14- and 18-fold, respectively (Fig. 3b), suggesting that these brain lipid extracts contain the putative specific lipid(s) for GlcT. The presence of PA and cholesterol (5%) in addition to egg PC (95%) increased the specific activity by 2.3- and 5.7-fold, respectively (Fig. 3b), indicating that these lipids are candidates of the specific lipids for GlcT. In contrast, syntheses in the presence of a bovine liver polar lipid extract or lyso PC (5%) or lyso PS (5%) and egg PC (95%) lipid mixtures exhibited no production (Fig. 3a, lanes “Liver polar ext.”, “PC + lyso-PC” and “PC + lyso-PS”).

For GlcT produced with the porcine brain polar lipid extract, we identified the lipid species included in the β-dodecyl-*D*-maltoside (βDDM)-solubilized, purified sample by the MS/MS analysis. Consequently, a hexosylceramide was detected in addition to the major lipids, PC(34:1) and PC(36:1) (Fig. 3c), and was presumed to be the major one in the brain lipid extract, galactosylceramide (GalCer). Accordingly, we produced GlcT with 5% GalCer and 5% cholesterol, which significantly increased the specific activity as described above, and egg PC (90%). This lipid combination drastically increased the GlcT production level, and higher total activity was concomitantly detected (Fig. 3d, lane “PC + GalCer + Chol”, and 3e). Therefore, GalCer, which is tightly bound to GlcT even after detergent solubilization, may also be one of the specific lipids for GlcT.

By contrast, the GlcT sample produced by the P-MF method with the porcine brain polar lipid extract exhibited 2.7-fold higher specific activity. Consequently, the different polar lipid lots probably had different compositions, and there must have been some component in the previous lot that resulted in the 14-fold increase, instead of the 2.7-fold increase. Disappointingly, the GlcT produced with the porcine brain polar lipid extract by the P-MF method was only poorly solubilized with detergent, and even the small ubiquitin-like modifier (SUMO)^23^-tagged GlcT, with solubility and stability enhanced by the fusion of a SUMO tag to the N-terminus of GlcT, was poorly solubilized (Fig. 3f). This may have occurred because the added lipids have properties related to detergent resistant membrane domain formation. Therefore, we tried the S-MF method, for comparison with the P-MF method.

**Figure 4:**
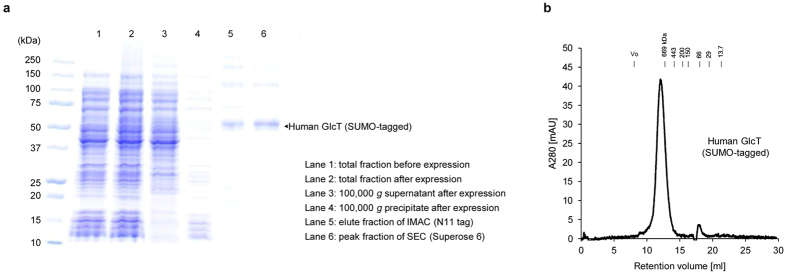
Purification of human GlcT by the S-MF method. (**a**) Purification of SUMO-tagged human GlcT by the S-MF method. Cell-free produced SUMO-tagged human GlcT in the 100,000 *g* supernatant was purified by SEC with a Superose 6 Increase column (**b**), after the His-affinity purification with the N11-tag on the N-terminus of GlcT. The purified samples were analyzed by SDS-PAGE with Coomassie Brilliant Blue staining. The experiment was performed once.

Using the S-MF method in the presence of the porcine brain polar lipid extract, human GlcT was produced well on a larger scale (Fig. 4a). In this case, the small membrane fragments are directly applicable to column chromatography, with no detergent solubilization step. Finally, we successfully purified human GlcT in the small membrane fragments to near homogeneity, by several chromatography steps (Fig. 4b). This is one of the significant advantages of the S-MF method.

### Human γ-secretase subunits

In cell-free synthesis, it is easy to coexpress the components of membrane protein complexes in their proper stoichiometry. Moreover, one (or more) of the components can be synthesized in the presence of other components that are prepared separately, and then added to the cell-free reaction solution. This method is useful for the expression of one or more components with low productivity, as sufficient amounts can be synthesized without competition with the other components in the cell-free translation, and/or the unstable component(s) may be folded properly and then stabilized by the other components.

**Figure 5:**
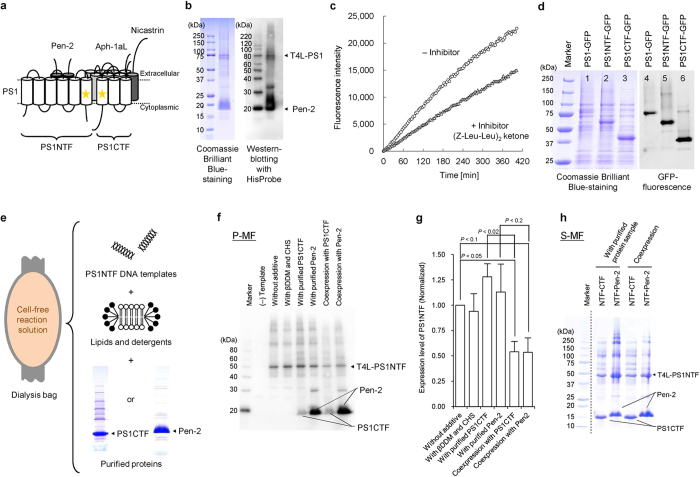
Production of the human γ-secretase components. (**a**) Schematic model of the γ-secretase complex. The stars on PS1 indicate the positions of the catalytic residues (D257 and D385 in human PS1). (**b**) SDS-PAGE images of the T4L-PS1∙Pen-2 complex, produced by the P-MF method and purified by the tag-affinity method with the FLAG-tag at the N-terminus of Pen-2, detected by Coomassie Brilliant Blue staining and western blotting with HisProbe. (**c**) The enzymatic activity of the T4L-PS1∙Pen-2 complex with the intramolecularly quenched fluorogenic peptide probe. The fluorescence intensities with (triangles) and without (circles) the GXGD protease inhibitor, (Z-Leu-Leu)_2_ ketone, at 0–420 min were plotted in this graph. (**d**) Rapid protein-folding assay, using GFP for the cell-free production of PS1 and the PS1NTF and PS1CTF fragments. The GFP fluorescence was observed with an ImageQuant LAS4000 system (GE Healthcare Life Sciences). (**e**) Conceptual diagram of the cell-free production of PS1NTF with the addition of purified PS1CTF or Pen-2 to the cell-free reaction solution. (**f**,**g**) Cell-free production of T4L-PS1NTF by the P-MF method, with either the coexpression of PS1CTF/Pen-2 or the addition of the purified proteins. (**f**) Cell-free-produced proteins in the membrane fractions were detected by western blotting with HisProbe-HRP. (**g**) Densitometry analysis of the bands of the cell-free-produced T4L-PS1NTF in (**f**) with means ± S.D. (error bars); *n* = 3. (**h**) Cell-free production of T4L-PS1NTF by the S-MF method, with either the coexpression of PS1CTF/Pen-2 or the addition of the purified proteins. The T4L-PS1NTF∙PS1CTF complex was purified with the FLAG-tag at the C-terminus of PS1CTF, and the T4L-PS1NTF∙Pen-2 complex was purified with the StrepII-tag at the C-terminus of Pen-2. The purified samples were detected by SDS-PAGE and Coomassie Brilliant Blue staining. Gel images are representative of at least two experiments, except for (**d**). All gel images in this figure were processed by cropping.

A representative example is the human γ-secretase complex, an intramembrane protease that generates amyloid-β peptide associated with Alzheimer’s disease^24^. This huge membrane protein complex (*ca*. 170 kDa) consists of four subunits (Fig. 5a). The catalytic nine-transmembrane presenilin-1 (PS1) subunit is considered to interact with the two-transmembrane presenilin enhancer protein 2 (Pen-2)^25^. PS1, fused at its N-terminus to T4 lysozyme for productivity improvement (T4L-PS1)^26^, and Pen-2 were co-produced from the two template DNAs by the P-MF method, and actually formed a complex (Fig. 5b). Furthermore, the full-length form of nicastrin, the single-transmembrane substrate-recognition subunit, was synthesized by the conventional method, and purified by affinity and gel-filtration chromatography (Supplementary Fig. S1c). By the P-MF method, the seven-transmembrane scaffold protein Aph-1aL (Fig. 5a) was produced with its N-terminus fused to SUMO, to a level detectable by Coomassie Brilliant Blue staining (Supplementary Fig. S1d).

The T4L-PS1∙Pen-2 complex exhibited protease activity and sensitivity to a GXGD protease inhibitor, (Z-Leu-Leu)_2_ ketone (Fig. 5c). However, the full-length PS1 tends to aggregate and proteolytically fragment (Fig. 2b, lane 19, Fig. 5b). Therefore, we coexpressed the N-terminal six-transmembrane PS1NTF (residues 1–292) and the C-terminal three-transmembrane PS1CTF (residues 379–467), which mimics the autolytic removal of the large flexible cytoplasmic loop region between helices 6 and 7 (Fig. 5a), to form the activated form of PS1. Actually, a similar activated form of a PS1 homolog was used for its crystal structure analysis^27^.

Various detergents were tested to identify those that facilitated the formation of the PS1NTF∙PS1CTF complex by coexpression of the component proteins. For this purpose, the S-MF method was used because the PS1NTF∙PS1CTF complex can be purified quickly and efficiently, without detergent solubilization. Thus, several favorable detergents, including Brij-78 and digitonin, were identified (Supplementary Fig. S2a). Brij-78 and digitonin were then tested, and the latter was superior to the former, with respect to the formation of the PS1NTF∙PS1CTF∙Pen-2 complex in the coexpression by the S-MF method (Supplementary Fig. S2b). Moreover, the purified PS1NTF∙PS1CTF∙Pen-2 complex prepared with digitonin showed higher protease activity than that prepared with Brij-78 (Supplementary Fig. S2c). In this context, the protease activity of the γ-secretase complex was observed with digitonin^28^.

Next, we checked if PS1NTF and PS1CTF could each properly fold by themselves, by a folding interference assay with green fluorescent protein (GFP)^29^,^30^. PS1NTF and PS1CTF, with GFP fusions at their C termini, were produced by the P-MF method (Fig. 5d). The PS1NTF-GFP and PS1CTF-GFP fusion proteins exhibited thick bands, which were clearer than that of the full-length PS1-GFP fusion protein (Fig. 5d, lane 1), in the Coomassie Brilliant Blue-stained PAGE gel. These thick bands presented strong fluorescence due to GFP (Fig. 5d, lanes 5 and 6), indicating that the two fusion proteins were well folded and did not interfere with the folding of the fluorescent GFP. On the basis of this observation, Pen-2 and PS1CTF were separately produced by the conventional cell-free method and purified (Supplementary Fig. S1a and b). PS1NTF was then synthesized by the cell-free P-MF method in the presence of either the Pen-2 or PS1CTF protein, in order to increase the production levels of PS1NTF in the forms of the PS1NTF∙Pen-2 and PS1NTF∙PS1CTF complexes, respectively (Fig. 5e). This complex formation procedure did not affect the PS1NTF synthesis level, due to the absence of competition with the synthesis of the binding partner, and high production levels of the complexes were achieved (Fig. 5f,g). These complexes were also produced by the cell-free synthesis of PS1NTF with the S-MF method in the presence of the purified proteins, and were purified with the affinity tags on Pen-2 and PS1CTF, in the same manner as that for coexpression (Fig. 5h). Experiments to form the complete γ-secretase complex are now in progress, using these cell-free products. These examples aptly demonstrate the power and flexibility of cell-free production, to achieve the proper formation of complexes containing multiple membrane protein components.

### Human claudin-family proteins

**Figure 6:**
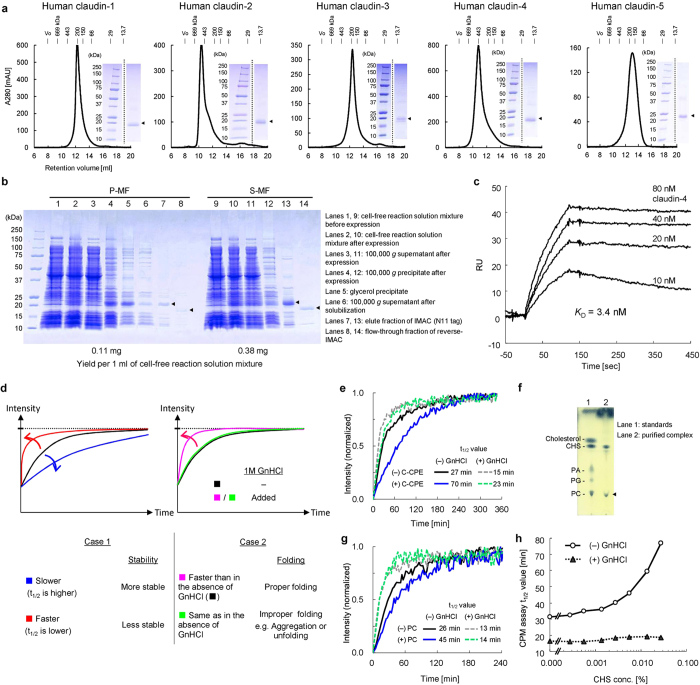
Properties of human claudins produced by the S-MF method. (**a**) SEC elution profiles of human claudins, prepared by the S-MF method. The peak fractions of SEC were analyzed by SDS-PAGE and Coomassie Brilliant Blue staining. (**b**) Comparison of the properties of claudin-4 produced by the P-MF and S-MF methods (lanes 1–8 and 9–14, respectively). The fractions in the purification steps were analyzed by SDS-PAGE and Coomassie Brilliant Blue staining. (**c**) SPR binding analysis of the cell-free produced claudin-4 against the immobilized GST-tagged C-CPE on the sensor chip. (**d**) Conceptual diagram of the thermal stability and folding analysis of a membrane protein by the CPM assay. (**e**) Thermal stability analysis of claudin-4 (open symbols) and the claudin-4∙C-CPE complex (closed symbols) by the CPM assay, in the absence (circles) or presence (triangles) of 1 M guanidine-HCl (GnHCl) at 40 °C. (**f**) TLC analysis for the detection of the lipids bound to claudin-4 in the purified claudin-4∙C-CPE complex sample. (**g**) The CPM assay for the assessment of the thermal stabilization effect of PC for the claudin-4∙C-CPE complex. (**h**) The CPM assay for the assessment of the thermal stabilization effect of CHS for the claudin-4∙C-CPE complex. The t_1/2_ values in the absence (open circles) and presence (closed triangles) of 1 M GnHCl at each concentration were plotted in this graph. The gel images and graphs are representatives of at least two experiments ((**a**–**c**), (**e**,**f**)) or one experiment (**g**,**h**). The gel images of (**a**) were processed by cropping.

The claudin-family four-transmembrane proteins are the major components of tight-junction strands, and are responsible for paracellular permeability and barrier function. In cells^31^, claudins are localized to detergent resistant membrane domains, and are only poorly solubilized with mild detergents^32^. Accordingly, it was challenging to produce a large amount of a purified claudin sample for X-ray crystallography^33^. In the present study, we first produced the human claudin-1 and claudin-4 proteins by the P-MF method, and obtained excellent production levels (Fig. 2a–c, lanes 15 and 16). We compared the P-MF and S-MF methods for the cell-free production of human claudin-4. First, claudin-4, N-terminally fused with the N11 affinity tag, was produced by the P-MF method. After solubilization with βDDM, the product was purified by the tag affinity method. In parallel, the type and concentration of detergent were examined for the cell-free production of claudin-4 by the S-MF method (Supplementary Fig. S3). After further optimization of the conditions, the N11-tagged claudin-4 in the solubilized form was produced by the S-MF method, and was directly tag purified. As shown in Fig. 6b, the translation levels by the P-MF and S-MF methods were the same (Fig. 6b, lanes 2 and 10). The membrane fraction containing claudin-4 was purified well by glycerol density gradient fractionation (Fig. 6b, lanes 4 and 5). However, the solubilization efficiency with βDDM was low, and most of the produced claudin-4 was lost (Fig. 6b, lane 6). In contrast, the solubilization process is not required in the S-MF method (Fig. 6b, lane 11). The final yields of the tag affinity-purified claudin-4 per milliliter cell-free synthesis reaction were 0.11 mg by the P-MF method and 0.38 mg by the S-MF method (Fig. 6b, lanes 8 and 14). Moreover, by using the claudin-4 sample prepared by the S-MF method, a monoclonal antibody that recognizes the tertiary structure of claudin-4 was successfully obtained (to be published elsewhere). The structural integrity of the claudin-4 produced by the S-MF method was higher than that generated by the conventional method (Supplementary Fig. S4a–c). In addition, the yield of claudin-4 by the S-MF method with egg PC + digitonin was higher than that with DMPC + DHPC, known as the bicelle^12^ (Supplementary Fig. S5a–c). Therefore, the S-MF method was suitable for the production of claudin-4 for X-ray crystallography.

In similar manners to that for claudin-4, we produced the human claudin-1, claudin-2, claudin-3, and claudin-5 proteins. All five claudin samples were synthesized at high levels by the S-MF method, and were purified through Ni-affinity chromatography. All of the cell-free products obtained by the S-MF method were monodisperse with minimal aggregation, as revealed by the size-exclusion chromatography (SEC) analysis (Fig. 6a). The molecular masses corresponding to the peak positions indicated that claudin-2 and claudin-4 each formed multimers. Therefore, the present strategy is expected to be applicable to many of the claudin family members.

### Interaction of human claudin-4 with *Clostridium perfringens* enterotoxin (CPE)

The food-poisoning bacterium *Clostridium perfringens* secretes an enterotoxin (CPE), which targets human claudin-4 with extremely high affinity^34^. We used surface plasmon resonance to measure the affinity of our claudin-4 sample, prepared by the S-MF method, for the C-terminal fragment of CPE, consisting of residues 185–319 (C-CPE), which contains the putative claudin-4 binding site. The measured *K*_D_ value was 3.4 nM (Fig. 6c), which is actually almost the same as that obtained by a cell-based assay^33^.

As shown in the conceptual diagram (Fig. 6d), the structural integrity and stability of the purified membrane protein can be evaluated by the 7-diethylamino-3-(4-maleimidophenyl)-4-methylcoumarin (CPM) assay. In this assay, the thermal denaturation of the analyte at 40 °C is monitored by the fluorescence of CPM dye, which reacts with exposed cysteine residues, if present. When the t_1/2_ value of thermal denaturation at 40 °C is 17 min or longer, the membrane protein is considered to be sufficiently stable^35^. In theory, a more stable membrane protein has a longer t_1/2_ value. However, the rigid aggregation of membrane proteins due to misfolding also increases the t_1/2_ value. Consequently, to distinguish between the desired stability and the undesired aggregation, we compare the CPM assays of a membrane protein in the absence and presence of 1 M guanidine-HCl (GnHCl). The properly folded protein exhibits a shorter t_1/2_ value in the presence of 1 M GnHCl (Fig. 6d, case 1), whereas the rigid aggregate does not show such a difference (Fig. 6d, case 2). Therefore, it is now possible to conclusively evaluate the status of the purified sample and the effects of the stabilizing factors. Consequently, the structural integrity and stability of the purified human claudin-4 were examined by the CPM assay.

The t_1/2_ values of the claudin-4 preparation in the absence and presence of GnHCl were 27 and 15 min, respectively, at 40 °C (Fig. 6e, gray solid and dashed lines, respectively), indicating that this sample has a sufficiently stable and integral structure. Furthermore, the t_1/2_ values of the complex of claudin-4 with C-CPE were 70 and 23 min in the absence and presence, respectively, of GnHCl (Fig. 6e, black solid and dashed lines, respectively). We then screened possible stabilizing factors, including the boundary lipids, by the CPM assay. The TLC analysis revealed that the claudin-4∙C-CPE complex sample contains PC (Fig. 6f), suggesting that PC is the boundary lipid for claudin-4. Therefore, we added PC to the sample, and found that the t_1/2_ value increased 1.7-fold (Fig. 6g, black solid line). In addition, a cholesterol analogue, cholesteryl hemisuccinate (CHS), increased the stability in a dose-dependent manner (Fig. 6h, white circles). By including PC and CHS in the production and purification of claudin-4, a higher yield was achieved. These two stabilizing factors were also used in the crystallization step, as described below.

### Crystallization and structure determination of the human claudin-4∙C-CPE complex

We performed a crystallization screening of the claudin-4∙C-CPE complex, and successfully obtained crystals diffracting to about 4-Å resolution (Supplementary Fig. S6a–c). The initial phase was determined by the molecular replacement – single-wavelength anomalous dispersion method, using the crystal structure of CPE^36^. The electron density map revealed the overall structure of the claudin-4∙C-CPE complex (Supplementary Fig. S6d). However, the crystal packing on the cytoplasmic side was worse than that on the extracellular side, and was insufficient for model building.

**Figure 7:**
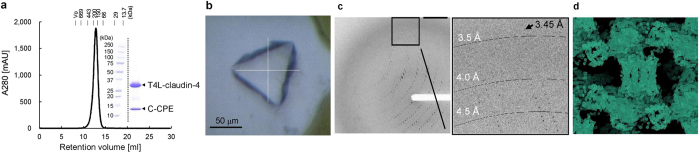
X-ray crystallography of the T4L-claudin-4∙C-CPE complex. (**a**) SEC elution profile of the T4L-claudin-4∙C-CPE complex. (**b**) The crystal of the T4L-claudin-4∙C-CPE complex. (**c**) The diffraction images collected from the crystal of the T4L-claudin-4∙C-CPE complex, using BL41XU of SPring-8. (**d**) The electron density map with the initial phase of the T4L-claudin-4∙C-CPE complex at 4.2-Å resolution. The maps were drawn using the program COOT^52^.

On the basis of the electron density map, we tried to improve the crystal packing by fusing T4 phage lysozyme (T4L)^37^ to the intracellular N-terminus of human claudin-4 (T4L-claudin-4). The T4L-claudin-4∙C-CPE complex was prepared in the same manner as the claudin-4∙C-CPE complex (Fig. 7a), and was similarly crystallized (Fig. 7b). The diffraction data set beyond 3.5-Å resolution was successfully collected at SPring-8 BL41XU (Fig. 7c). The experimental phase of this data set was determined by the molecular replacement – single-wavelength anomalous dispersion method, using the coordinates of CPE and T4L^21^,^37^. In fact, the electron density was greatly improved (Fig. 7d), as compared to that shown in Supplementary Fig. S6d. All of these crystallographic results unambiguously demonstrated that the membrane protein samples produced by the S-MF method are highly purified and structurally intact. The refined crystal structure of the T4L-claudin-4∙C-CPE complex will be published elsewhere.

## Discussion

The cell-free methods are expected to become the standard procedures for crystallography-oriented membrane protein preparation, as they overcome a number of difficulties that unavoidably accompany the cell-based recombinant expression methods with respect to the preparation of mammalian membrane proteins. These expectations have been mentioned previously^38^,^39^, but have not been met successfully in the crystallography of mammalian membrane proteins at atomic resolution. In this study, we applied the P-MF and S-MF methods to the cell-free protein production and purification of mammalian α-helical membrane proteins. We thereby successfully performed the first X-ray crystallographic analysis of a mammalian membrane protein prepared by the cell-free method.

In the conventional method (Fig. 1), detergents, but not lipids, are included in the *E. coli* cell-free synthesis solution^6^. The synthesized membrane proteins are harvested as proteomicelles in the 100,000 *g* supernatant. Using this conventional method, the protein synthesis yield is usually higher, but the conformational homogeneity and stability of the product are generally worse, as compared to those of the proteins produced by the P-MF and S-MF methods.

By the P-MF method, the synthesized membrane protein is integrated efficiently into the lipid bilayers of large membrane fragments. In order to achieve the functional and structural integrity of the membrane protein, the conditions must be optimized, by selecting the bound-lipid components and the binding-partner proteins, for addition to both the purification buffer and cell-free production solution. The membrane protein produced by the P-MF method is present in large membrane fragments and does not require the preparation of solubilized samples, and thus can be tested for the effects of lipids *etc*. on the production yield, the function (*e*.*g*. enzymatic activity), and the structure (*e*.*g*. folding). Thus, in the optimized lipid bilayer environment, the membrane protein may be produced in a form similar to that in the cell membrane.

On the one hand, the optimized, cell membrane-like lipid bilayer environment of the cell-free production may cause some difficulty with detergent solubilization, as found for GlcT and claudin-4. In this context, when the membrane protein is produced by the cell-based recombinant method, the expressed membrane protein tends to be resistant to solubilization with mild detergents, and to lose its structural and functional integrity upon solubilization with harsh detergents from the cell membrane. This problem is, at least partially, reproduced in the cell-free production.

On the other hand, by the S-MF method, the synthesized membrane protein is integrated efficiently into the lipid bilayers of small membrane fragments. By applying the optimized conditions; *i*.*e*., the presence of bound-lipid components and/or binding-partner proteins, revealed by the P-MF method, it is possible to obtain a product with functional and structural integrity in the environment of small membrane fragments. As the S-MF product is collected in the 100,000 *g* supernatant, solubilization with harsh detergents is unnecessary. Therefore, the small membrane fragments containing the membrane protein can be directly subjected to column chromatography, such as tag-affinity chromatography and SEC. When the cell-free product is monodisperse and possesses structural integrity and stability, the SEC elution peak exhibits a sharp symmetrical shape. The structural integrity and stability of the product can also be confirmed by a CPM assay and/or a TLC analysis of the bound lipids. Consequently, the yield of crystallizable membrane protein prepared by the S-MF method is appreciably higher than that by other cell-free methods. Finally, it should be emphasized that the high quality membrane protein produced by the present cell-free method can be used not only for X-ray crystallography, but also for general biological experiments, drug evaluations, antibody preparation as the immunogen, and other purposes.

## Methods

### Materials

Digitonin was purchased from Wako Pure Chemical Industries, cholesteryl hemisuccinate (CHS) and most of the detergents were from Anatrace, Brij-35, Brij-58, Brij-78, cholesterol, 3-*sn*-phosphatic acid (PA), L-α-phosphatidylethanolamine (PE), L-α-phosphatidylinositol (PI), L-α-phosphatidyl-L-serine (PS), 3-[(3-cholamidopropyl)dimethylammonio]-1-propanesulfonate (CHAPS) and 7-diethylamino-3-(4-maleimidophenyl)-4-methyl coumarin (CPM) were from Sigma-Aldrich, L-α-lysophosphatidylethanolamine (lyso-PE), L-α-lysophosphatidylserine (lyso-PS), and L-α-lysophosphatidylcholine (lyso-PC) were from Olbracht Serdary Research Laboratories, 1,2-dimyristoyl-*sn*-glycero-3-phosphocholine (DMPC) and natural lipids were from Avanti Polar Lipids, and pentaethylene glycol decyl ether (C_10_E_5_), octaethylene glycol decyl ether (C_10_E_8_), hexaethylene glycol dodecyl ether (C_12_E_6_), and octaethylene glycol dodecyl ether (C_12_E_8_) were from Nikko Chemicals. The intramolecularly-quenched fluorogenic peptide probe, Nma-Gly-Gly-Val-Val-Ile-Ala-Thr-Val-Lys(Dnp)-D-Arg-D-Arg-D-Arg-NH_2_, and the GXGD protease inhibitor, (Z-Leu-Leu)_2_ ketone, were purchased from Peptide Institute, Inc., and the fluorescent acceptor substrate for GlcT, C6-nitrobenzoxadiazole (NBD)-ceramide, and the GlcT inhibitor, D-*threo*-1-phenyl-2-decanoylamino-3-morpholino-1-propanol (PDMP), were obtained from Cayman Chemical and Matreya, respectively. Reagents and tools for AlphaScreen were purchased from PerkinElmer, and those for X-ray crystallography were from Hampton Research and Molecular Dimensions. Precast gradient polyacrylamide gels, XV PANTERA Gel 10–20%, were purchased from DRC. The cDNA clones encoding P07550, P25101, P25024, P32248, P34998, P19438, P42262, P22001 and O43315 were purchased from OriGene Technologies Inc., Q9GZQ4, P57739 and O15551 were from Invitrogen, Q9UBL9 was from GeneCopoeia, and O14493 was from Research Association for Biotechnology. All other chemicals were purchased from Nacalai Tesque.

### Cell-free production trial of mammalian membrane proteins using the *E. coli* cell-free protein synthesis system under the initial conditions

The expression vector for the mammalian membrane proteins, N-terminally fused with a modified natural poly-histidine (N11, MKDHLIHNHHKHEHAHAEH) affinity tag, a TEV protease recognition site, and the linker sequence GSSGSSG^40^, was constructed by TOPO cloning (Life Technologies, USA). Cell-free synthesis of the membrane proteins was performed with the *E. coli* S30 extract by the P-MF method^13^. The *E. coli* S30 extract was prepared from *E. coli* BL21(DE3) cells bearing the pMINOR plasmid^5^ in S30 buffer [10 mM Tris acetate buffer (pH 8.2), containing 60 mM potassium acetate, 16 mM Mg(OAc)_2_, and 1 mM DTT]. Mixed micelles were prepared from a mixture of 50 mg/ml egg PC liposome and 30 mg/ml digitonin, by sonication with a VP-30S sonicator (TAITEC) until the solution became clear. The reaction solution was prepared with 0.37 volume of the low-molecular weight creatine phosphate tyrosine (LMCPY) mixture [160 mM HEPES-KOH buffer (pH 7.5), containing 4.13 mM tyrosine, 3.47 mM ATP, 2.40 mM each of GTP, CTP, UTP, 0.217 mM folic acid, 1.78 mM cAMP, 74 mM ammonium acetate, 214 mM creatine phosphate, 5 mM DTT, 534 mM potassium L-glutamate, and 10.7% PEG8000], 0.075 volume of the 19-amino acid mixture [20 mM each of the amino acids other than L-tyrosine], 0.01 volume of 5% NaN_3_, 0.0057 volume of 1.6 M Mg(OAc)_2_, 0.067 volume of 3.75 mg/ml creatine kinase, 0.01 volume of 17.5 mg/ml tRNA, 0.0067 volume of 10 mg/ml T7 RNA polymerase, 0.3 volume of *E. coli* S30 extract, 0.033 volume of 120 μg/ml template plasmid, and 0.13 volume of the mixed micelles, in that order. The external solution was prepared with 0.37 volume of the LMCPY mixture, 0.075 volume of the 19-amino acid mixture, 0.01 volume of 5% NaN_3_, 0.0057 volume of 1.6 M Mg(OAc)_2_, and 0.3 volume of the S30 buffer. The 30-μl reaction solution was dialyzed against 1-ml external solution at 30 °C for 5 hr with shaking, and then centrifuged at 100,000 *g* at 4 °C for 30 min. Thus, the membrane fraction containing the synthesized membrane protein was collected as the 100,000 *g* precipitate. The production level of each reaction was examined by SDS-PAGE, Coomassie Brilliant Blue staining, and western blotting with HisProbe-HRP (Pierce). Densitometry of the cell-free-synthesized membrane protein bands in the SDS-PAGE gels was performed using ImageJ (http://imagej.nih.gov/ij/). The amounts of proteins were estimated from the calibration curve, using bovine serum albumin (BSA) as the standard.

### Preparation of human GlcT by the P-MF method

Human GlcT was produced with various lipids by the P-MF method (see Fig. 3a,d). Cell-free protein synthesis was performed with mixed micelles of 3.3–6.7 mg/ml lipid and 2.0–4.0 mg/ml digitonin in the reaction solution without PEG8000. After dialysis against the external solution without PEG8000 at 25 °C for 5 hr, the reaction solution was centrifuged at 100,000 *g*. The precipitate, containing membrane-embedded GlcT, was fractionated by glycerol density gradient centrifugation at 100,000 *g* for 2 hr, with a 0–30% (w/w) glycerol gradient prepared in 50 mM MES-NaOH buffer (pH 6.0). The resultant pellet was then resuspended in 50 mM MES-NaOH buffer (pH 6.0), containing 400 mM NaCl. In the purification, the GlcT protein was extracted from membranes with 50 mM MES-NaOH buffer (pH 6.0), containing 1% βDDM, 0.2% CHS, 2 mM UDP-glucose, and 400 mM MgCl_2_, and was centrifuged at 100,000 *g* for 30 min at 4 °C. The solubilized GlcT protein in the supernatant was adsorbed on TALON resin (Clontech), which was washed with 25 column volumes of wash buffer [50 mM MES-NaOH buffer (pH 6.0), containing 0.05% βDDM, 0.002% CHS, 400 mM NaCl, 5 mM MgCl_2_, and 2 mM UDP-glucose], and then eluted with 20 column volumes of elution buffer [50 mM MES-NaOH buffer (pH 6.0), containing 200 mM imidazole, 0.05% βDDM, 0.002% CHS, 400 mM NaCl, 5 mM MgCl_2_, and 2 mM UDP-glucose]. The protein-bound lipids in the purified fraction of GlcT were extracted by the Bligh and Dyer method^41^, and analyzed by ESI-MS using a 4000Q TRAP triple quadrupole mass spectrometer (AB SCIEX). The mobile phase was 5 mM ammonium formate in methanol with 0.2% formic acid, at a flow rate of 100 μl/min. The MS/MS analysis was performed in the positive ion mode with a collision energy of 30 eV, and lipid species were identified as described^42^,^43^,^44^. The enzymatic activity of GlcT was assayed with C6 NBD-ceramide as the substrate, according to the previously described method^22^.

### Preparation of SUMO-tagged human GlcT by the S-MF method

The N11-SUMO-tagged human GlcT without the TEV site (SUMO-GlcT) was produced by the S-MF method. Mixed micelles were prepared by sonicating a mixture of 25 mg/ml liposomes (porcine brain polar lipid extract) and 75 mg/ml digitonin, until the solution became clear. The reaction solution contained 0.13 volume of the mixed micelles and all of the other components described above for the P-MF method, except PEG8000. The reaction solution was dialyzed against the external solution without PEG8000 at 25 °C for 5 hr, and was centrifuged at 100,000 *g*. The 100,000 *g* supernatant, containing SUMO-GlcT, was adsorbed onto Ni-Sepharose resin (GE Healthcare Life Sciences), which was washed with 20 column volumes of wash buffer [50 mM Tris-HCl buffer (pH 7.0), containing 0.05% βDDM, 0.002% CHS, 400 mM NaCl, 5 mM MgCl_2_, and 2 mM UDP-glucose], and then eluted with 5 column volumes of elution buffer [50 mM Tris-HCl buffer (pH 7.0), containing 400 mM imidazole, 0.05% βDDM, 0.002% CHS, 400 mM NaCl, 5 mM MgCl_2_, and 2 mM UDP-glucose]. The eluted fractions were pooled and immediately diluted with an equal volume of SEC running buffer [50 mM Tris-HCl buffer (pH 7.0), containing 0.05% βDDM, 0.002% CHS, 400 mM NaCl, 5 mM MgCl_2_], to prevent the eluted SUMO-GlcT from aggregating. The aggregates were removed by SEC on a HiLoad 16/60 Superdex 200 column (GE Healthcare Life Sciences). The SUMO-GlcT was finally separated into a mono-disperse peak by SEC on a Superose 6 Increase 10/300 column (GE Healthcare Life Sciences).

### Purification of the cell-free produced human γ-secretase components, PS1, Pen-2, Aph-1aL, nicastrin, and their mutants

The genes encoding the subunits of the human γ-secretase complex, except for Aph-1aL, were cloned into the same expression vector as described above. The gene encoding human Aph-1aL was fused N-terminally with the N11-SUMO tag and C-terminally with the TEV protease recognition site and the FLAG tag. The T4 phage lysozyme-human PS1 fusion protein (T4L-PS1)^26^ was produced by the P-MF method, with mixed micelles (3.3 mg/ml total or polar extract of porcine brain and 2.0 mg/ml digitonin) and without PEG8000. The proteoliposome fractionation, the membrane extraction, and the metal affinity purification were performed as described in the previous section, with some modifications: the use of 50 mM Tris-HCl buffer (pH 7.0) for the glycerol density gradient, 50 mM Tris-HCl buffer (pH 7.0) containing 1% βDDM, 0.1% CHS, and 400 mM NaCl for membrane extraction, Ni-Sepharose resin (GE Healthcare Life Sciences), the wash buffer [50 mM Tris-HCl buffer (pH 7.0) containing 0.05% βDDM, 0.002% CHS and 400 mM NaCl] and the elution buffer [50 mM Tris-HCl buffer (pH 7.0) containing 0.05% βDDM, 0.002% CHS, 200 mM imidazole, and 400 mM NaCl] for metal-affinity purification. The enzymatic activity of PS1 was assayed by the method using an intramolecularly-quenched fluorogenic peptide probe, Nma-Gly-Gly-Val-Val-Ile-Ala-Thr-Val-Lys(Dnp)-D-Arg-D-Arg-D-Arg-NH_2_^45^.

### Cell-free production of T4L-PS1NTF in the presence of purified PS1CTF or Pen-2

PS1CTF and Pen-2 were synthesized by the conventional method with 1% Brij-78 and without PEG8000, and purified by chromatography on a His-affinity column (Ni-Sepharose) and a size-exclusion column (Superdex 200 10/300, GE Healthcare Life Sciences), in 50 mM Tris-HCl buffer (pH 7.0), containing 0.05% βDDM, 0.002% CHS, and 400 mM NaCl. For the cell-free production of T4L-PS1NTF by the P-MF method, 1–3 mg of purified PS1CTF or Pen-2 was added to 1 ml of the cell-free reaction solution.

### Cell-free production and purification of human claudin-4 and C-CPE

The gene encoding human claudin-4 (residues 1–183) was subcloned, as described above in the subsection “Cell-free production trial of mammalian membrane proteins using the *E. coli* cell-free protein synthesis system under the initial conditions”, except for the addition of the sequences encoding the TEV protease recognition site and the FLAG tag on the 3′ end. The C-terminal 26 residues of claudin-4 were deleted because the C-terminus of the cell-free synthesized protein appeared to be fragmented, as judged from a MALDI-TOF MS analysis (VOYAGER, Applied Biosystems). The T4 phage lysozyme-claudin-4 fusion protein (T4L-claudin-4) was generated as previously reported^26^, but the TEV site and the FLAG-tag were added on the C-terminal side. Claudin-4 and T4L-claudin-4 were produced by the S-MF method with PEG8000, in the presence of mixed micelles consisting of 6.7 mg/ml lipids (5% (w/w) cholesterol and 95% (w/w) egg PC) and 10.0 mg/ml digitonin at 30 °C for 5 hr. The selenium-labeled sample was prepared in the same manner as the native protein, except that the methionine in the reaction and external solutions was replaced with selenomethionine. The claudin-4 produced in the reaction solution was collected in the 100,000 *g* supernatant, adsorbed on Ni-Sepharose affinity resin, washed with wash buffer A [50 mM Tris-HCl buffer (pH 7.0), containing 0.05% βDDM, 0.002% CHS, 20 mM imidazole, and 400 mM NaCl], and then eluted with 500 mM imidazole in the same buffer. The C-CPE protein (residues 185–319 of CPE), fused with the N11- and FLAG-tags, the TEV protease recognition site, and the GSSGSSG linker sequence at its N-terminus, was prepared in the same manner as claudin-4, except for the absence of detergents and lipids. The N-terminal N11-tag of claudin-4 was cleaved by digestion with His-tagged TEV protease at 4 °C overnight, while dialyzing against dialysis buffer A [50 mM Tris-HCl buffer (pH 7.0), containing 0.05% βDDM, 0.002% CHS, and 400 mM NaCl]. The N11-tag fragment and the His-tagged TEV protease were removed by reverse immobilized metal-ion affinity chromatography (IMAC), and the tag-free claudin-4 was recovered in the flow-through and wash fractions. The resultant claudin-4 was mixed with an excess amount of purified C-CPE, and incubated at 4 °C for 1 hr to form the claudin-4∙C-CPE complex. The complex was adsorbed on Ni-Sepharose resin through the N11-tag of C-CPE, washed with wash buffer B [50 mM Tris-HCl buffer (pH 8.0), containing 0.025% C_12_E_8_, 0.002% CHS, and 150 mM NaCl], and eluted with elution buffer B [50 mM Tris-HCl buffer (pH 8.0), containing 0.025% C_12_E_8_, 0.002% CHS, 500 mM imidazole, and 150 mM NaCl]. After the purification of the complex, the N11-tag of C-CPE was cleaved by TEV protease digestion at 4 °C overnight, while dialyzing against dialysis buffer B [50 mM Tris-HCl buffer (pH 8.0), containing 0.025% C_12_E_8_, 0.002% CHS, and 150 mM NaCl]. The N11-tag fragment was removed as described above. Finally, the sample was fractionated on a size-exclusion column (Superdex 200 10/300) in running buffer [50 mM Tris-HCl buffer (pH 8.0), containing 0.025% C_12_E_8_ and 150 mM NaCl], and separated into a mono-disperse peak of the protein complex. Prior to crystallization, the purified complex was incubated with 0.05 mg of egg PC lipid per mg protein at 4 °C for 30 min, and then with 25 μl of Biobeads SM2 (Bio-Rad) per ml protein solution at 4 °C for 1 hr. The Biobeads were removed by filtration through a 0.22 μm filter (Millipore). The pretreated sample was concentrated to approximately 20 mg/ml with an Amicon Ultra-4 filter, with a 10,000-Da molecular weight cut-off (Millipore). The T4L-claudin-4∙C-CPE complex was prepared in the same manner as the claudin-4∙C-CPE complex.

### Crystallization and data collection of the claudin-4∙C-CPE complexes

The claudin-4∙C-CPE complex and the T4L-claudin-4∙C-CPE complex were both crystallized in similar manners. A 6–8 mg/ml pretreated and concentrated protein sample was incubated in 3.6–10.0 mg/ml of the lipid suspension, in 20 mM Tris-HCl buffer (pH 7.0), containing 0.75% C_12_E_8_ and 10 mM reduced glutathione; cholesterol/sphingomyelin/egg PC (7.5:2.5:90, w/w) and egg PC were used for the claudin-4∙C-CPE and T4L-claudin-4∙C-CPE complexes, respectively. After an incubation on ice for 1 hr, the sample was mixed with an equal volume of the reservoir solution: 75 mM MES-NaOH buffer (pH 5.5–6.0), containing 20% (w/v) PEG3350, 5–7% (v/v) 2-methyl-2,4-pentanediol, 0.002% (w/v) NaN_3_, 0.0005% (w/v) 2,6-di-*t*-butyl-*p*-cresol, and 150 mM NaCl for the claudin-4∙C-CPE complex, and 75 mM MES-NaOH buffer (pH 5.0–5.5), containing 20% (w/v) PEG3350, 7–10% (v/v) 1,6-hexanediol, 0.002% (w/v) NaN_3_, 0.0005% (w/v) 2,6-di-*t*-butyl-*p*-cresol, and 150 mM NaCl for the T4L-claudin-4∙C-CPE complex. Crystallization screening was performed by the hanging-drop vapor diffusion method at 15 °C. Crystals appeared after about 2 weeks, and grew to over 50 × 50 × 50 μm^3^ in around 1–2 months. The crystals were soaked in the reservoir solution supplemented with 10% (w/v) glycerol for 1 hr, mounted on a cryo-loop, and flash-cooled in cold nitrogen gas. The X-ray diffraction data of the crystals were collected using BL32XU^46^,^47^ or BL41XU at SPring-8, with the MX225HE CCD detector in the 100-K cryostream. The wavelength for SAD data collection was 0.9793 Å for both crystals. Approximately 240 diffraction images were collected from each crystal, and the image data were processed with HKL-2000^48^ or XDS^49^. The space group and the cell dimensions of the crystal of the claudin-4∙C-CPE complex were *C*2 and *a* = 126.9 Å, *b* = 46.5 Å, *c* = 160.4 Å, *β* = 111.4°, respectively, and those of the crystal of the T4L-claudin-4∙C-CPE complex were *P*4_3_ and *a* = *b* = 105.9 Å, *c* = 244.3 Å, respectively.

### Phasing of X-ray diffraction data

We first tried MR, using the coordinates of C-CPE^36^ (residues 205–319, PDB: 3AM2). Correct packing was determined with the program Phaser-MR^50^ in the PHENIX suite^51^, using the low-resolution dataset from the *P*4_3_ crystal of the complex of the native T4L-claudin-4 and the selenomethionine-substituted C-CPE at 4.2 Å resolution. Based on this MR solution, we tried MR again, using the coordinates of T4L from the β2-adrenergic receptor-T4L fusion protein^21^,^37^ (PDB: 3D4S and 2RH1). The electron density maps of the helices and the β strands of claudin-4 were visualized by the molecular replacement – single-wavelength anomalous dispersion method, using the program Phaser-EP^50^ with the MR solution including four C-CPE and four T4L molecules.

### Surface plasmon resonance (SPR) analysis

The SPR analysis was performed with a Biacore 3000 (GE Healthcare Life Sciences). The monoclonal mouse anti-GST IgG_2a_ κ (Nacalai Tesque, 7,000 RU) was immobilized on the sensor chip CM5 by amine coupling, according to the manufacturer’s instructions, and 200 RU of the GST-tagged C-CPE were fixed on the sensor chip *via* the immobilized anti-GST antibodies in a sample flow cell in running buffer [20 mM Tris-HCl buffer (pH 8.0), containing 0.05% βDDM, 0.0017% CHS, and 150 mM NaCl], at a flow rate of 20 μl/min. C-CPE was not fixed in the reference flow cell. The purified tag-free claudin-4 was serially diluted to 10, 20, 40 and 80 nM in the running buffer, and 40 μl portions of the diluted samples were injected, in the order of increasing concentrations, into the sample and reference flow cells. The *K*_D_ value was determined with the Biacore3000 evaluation software, using a 1:1 binding model for fitting to the sensorgrams.

### AlphaScreen

The purified claudin-4 samples were diluted to 40 nM (final 10 nM) in the reaction buffer [50 mM Tris-HCl buffer (pH 7.0), containing 0.05% βDDM, 0.002% CHS, and 400 mM NaCl], and 5 μl aliquots were dispensed per well in a 384-well plate (AlphaPlate-384, PerkinElmer) on ice. In the blank well, an equal volume of the reaction buffer was dispensed. The purified GST-tagged C-CPE was also diluted to 160 nM (final 40 nM) in the reaction buffer, and a 5 μl aliquot of diluted GST-tagged C-CPE was added to all wells, mixed by shaking 30 sec, and then incubated on ice. Two hours later, a 5 μl aliquot of 80 μg/ml (final 20 μg/ml) Glutathione acceptor beads (PerkinElmer) in the reaction buffer was added to all wells, mixed by shaking 30 sec, and then incubated on ice for 1 hr in the dark. Finally, a 5 μl aliquot of 80 μg/ml (final 20 μg/ml) Anti-FLAG donor beads (PerkinElmer) in the reaction buffer was added to all wells, mixed by shaking 30 sec, and then incubated on ice for 1 hr in the dark. Before measurement, the plate was incubated at room temperature for 20 min in the dark. Alpha counts were measured by EnVision (PerkinElmer), using the standard AlphaScreen settings.

### Thermal stability analysis of claudin-4 by the CPM assay

The CPM assay was performed by the procedure of Hanson *et al*.^21^, with modifications. Prior to the start of the reaction, a 100 μl aliquot of the reaction buffer [50 mM Tris-HCl buffer (pH 7.0 or 8.0), containing 0.05% βDDM, 0.02% CHS, and 150 mM NaCl, with or without 0.2 M guanidine-HCl (GnHCl) as a chemical denaturant] was dispensed per well in a 96-well black plate (Nunc), and was kept at 25 °C until the start of the measurement. The purified protein samples were diluted to 0.05 mg/ml in the reaction buffer, and 240 μl aliquots were dispensed per well in another 96-well plate on ice. In the blank well, an equal volume of the reaction buffer was dispensed. A 24 μl aliquot of 0.4 mg/ml CPM in the reaction buffer was added to all wells, mixed by pipetting 10 times, and then incubated on ice for 15 min. Aliquots (100 μl) of the mixtures were transferred to the wells in the black plate, and mixed by pipetting 10 times, and then the measurement was started immediately. The fluorescence with excitation at 355 nm and emission at 460 nm was measured at every minute for 4–6 hr at 40 °C, with an ArvoX3 fluorophotometer (PerkinElmer). The t_1/2_ value, defined as the time when the CPM fluorescence intensity reaches the half maximum, was calculated from the linear region near t_1/2_ in each fluorescence intensity curve.

### Thin-layer chromatography analysis of lipids bound to the purified claudin-4∙C-CPE complex

Lipids were extracted with chloroform/methanol (2:1, v/v) from 30 μg of the purified claudin-4∙C-CPE complex, and these lipid extracts were then spotted onto Silica Gel 60 TLC plates (Merck). The lipids were separated with a mobile phase including chloroform, methanol, *n*-hexane, diethyl ether, acetic acid, and water (75:45:80:20:13:6, v/v), and detected with a phosphomolybdic acid reagent (Merck).

### Statistics

Statistical analyses were performed using Welch’s t-test in Microsoft EXCEL.

## Additional Information

**Accession codes:** Swiss-Prot accession codes for the 19 mammalian membrane proteins are listed in Fig. 2. Human Pen-2, Q9NZ42; human Aph-1aL, Q96BI3; human nicastrin, Q92542; human claudin-2, P57739; human claudin-3, O15551; human claudin-5, O00501; CPE, Q0SVZ0.

**How to cite this article**: Shinoda, T. *et al*. Cell-free methods to produce structurally intact mammalian membrane proteins. *Sci. Rep.* **6**, 30442; doi: 10.1038/srep30442 (2016).

## Supplementary Material

Supplementary Information
