# Supplementary material for: Cell-free methods to produce structurally intact mammalian membrane proteins
Source: Sci Rep. 2016 Jul 28;6:30442. doi: 10.1038/srep30442 (PMC4964339; doi:10.1038/srep30442)
Supplement: Supplementary Information [file srep30442-s1.pdf]

Supplementary Information

Cell-free methods to produce structurally intact mammalian membrane proteins

Takehiro Shinoda[1,2], Naoko Shinya[1,2], Kaori Ito[1,2], Yoshiko Ishizuka-Katsura[1,2], Noboru Ohsawa[1,2], Takaho Terada[1,6], Kunio Hirata[3], Yoshiaki Kawano[3], Masaki Yamamoto[3], Taisuke Tomita[4], Yohei Ishibashi[5], Yoshio Hirabayashi[5], Tomomi Kimura-Someya[1,2], Mikako Shirouzu[1,2]* & Shigeyuki Yokoyama[1,6]*

[1]RIKEN Systems and Structural Biology Center, Yokohama 230-0045, Japan.

[2]Division of Structural and Synthetic Biology, RIKEN Center for Life Science Technologies, Yokohama 230-0045, Japan.

[3]RIKEN SPring-8 Center, 1-1-1, Kouto, Sayo-cho, Sayo-gun, Hyogo 679-5148, Japan.

[4]Department of Neuropathology and Neuroscience, Graduate School of Pharmaceutical Sciences, The University of Tokyo, Bunkyo-ku, Tokyo 113-0033, Japan.

[5]Laboratory for Molecular Membrane Neuroscience, RIKEN Brain Science Institute, Wako, Saitama 351-0198, Japan.

[6]RIKEN Structural Biology Laboratory, Yokohama 230-0045, Japan.

*Correspondence should be addressed to M. S. (mikako.shirouzu@riken.jp) or S. Y. (yokoyama@riken.jp)

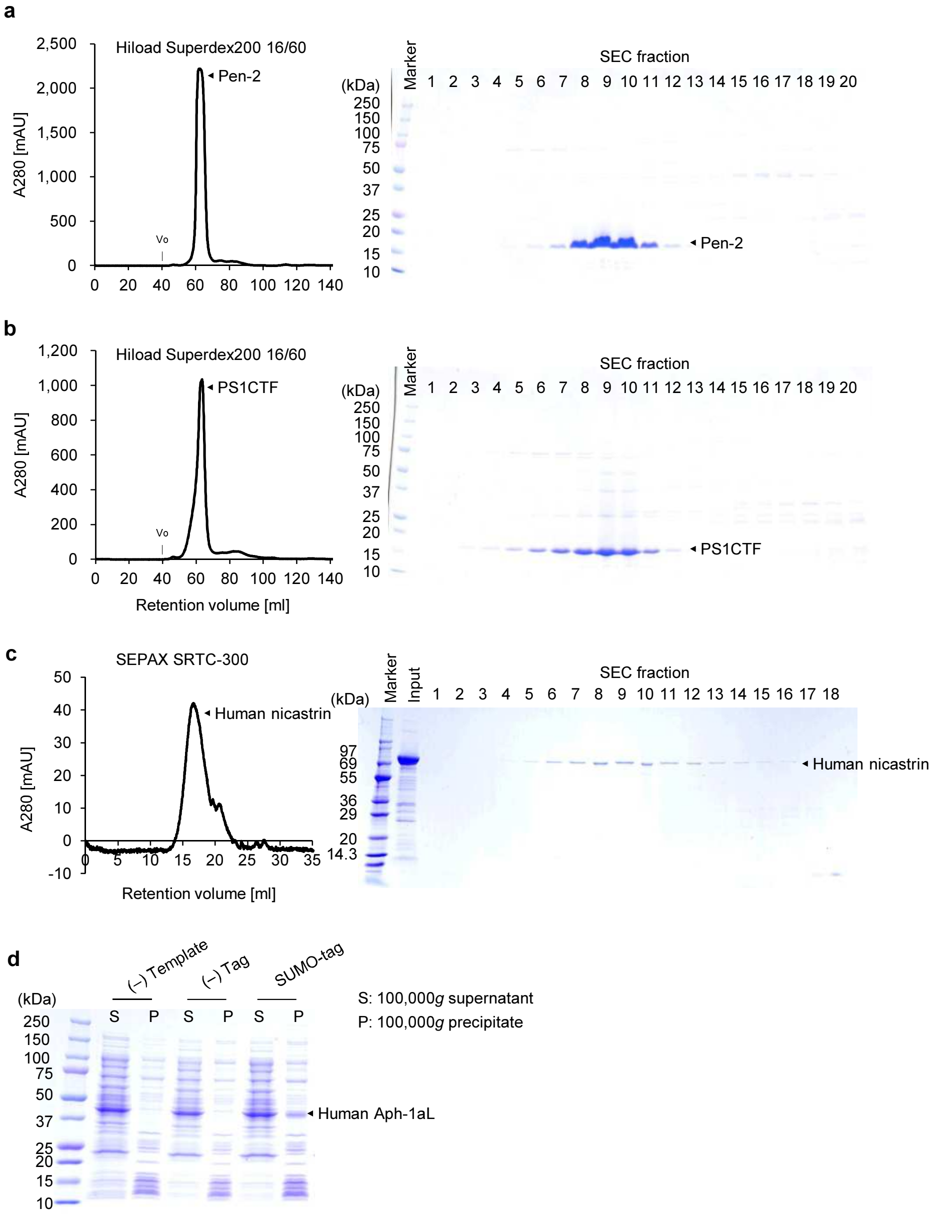

Supplementary Figure S1 | Cell-free production of the human γ-secretase components: PS1CTF, Pen-2, nicastrin and Aph-1aL. (a)–(c) SEC profiles of purified human Pen-2 (a), PS1CTF (b) and nicastrin (c), produced by the conventional method. Cell-free produced human PS1CTF and Pen-2 were purified by His-affinity chromatography with the N11-tags at their N-termini and by SEC. In the SEC elution profile, the membrane proteins were eluted as proteomicelles, before the contaminating soluble proteins with larger molecular masses. Cell-free produced human nicastrin was purified by SEC, after FLAG-affinity chromatography and anion-exchange chromatography. (d) Comparison of the production levels of Aph-1aL with and without the N-terminal SUMO-tag by the P-MF method. Each SDS-PAGE gel was visualized by Coomassie Brilliant Blue staining.

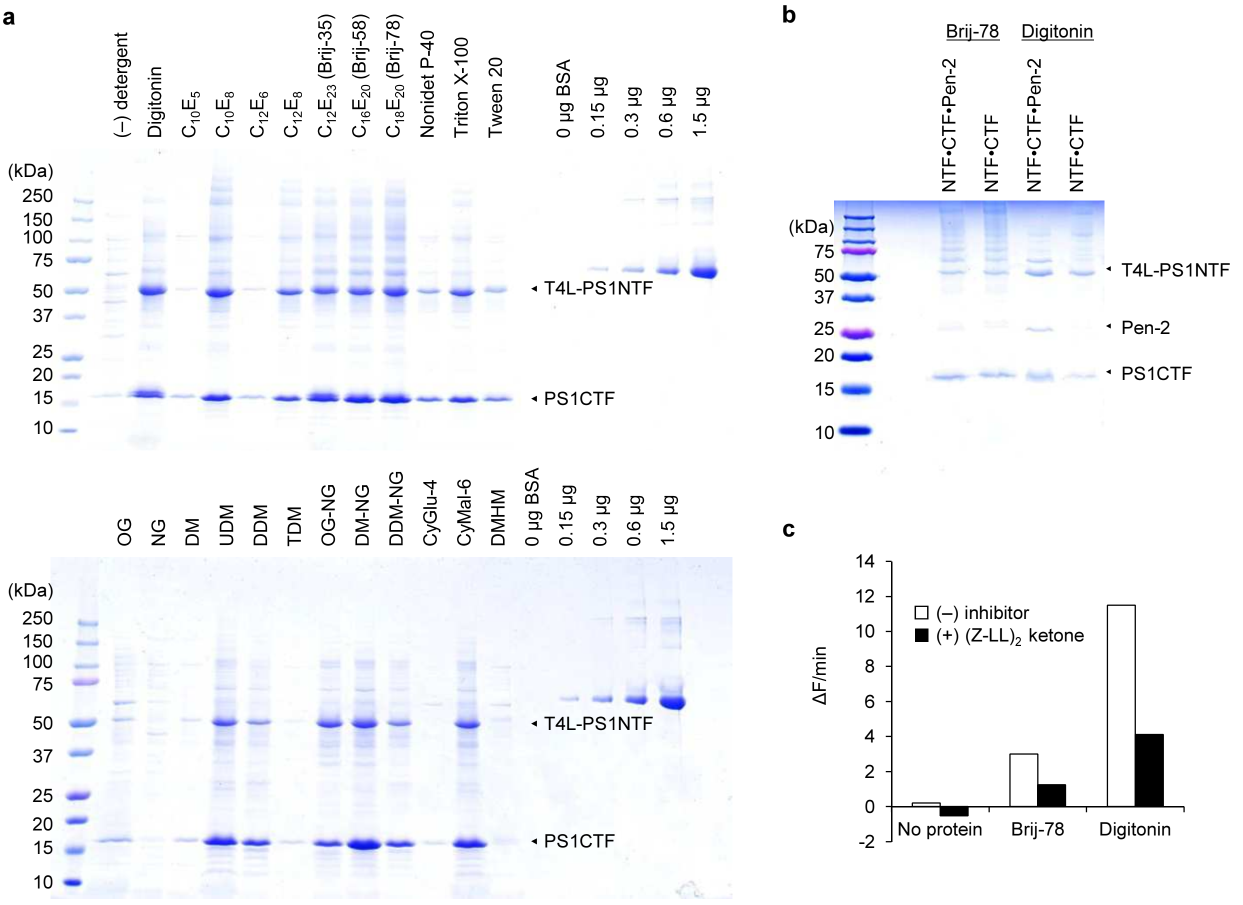

Supplementary Figure S2 | Production of the human γ-secretase components by the S-MF method. (a) SDS-PAGE images of the T4L-PS1NTF•PS1CTF complex, produced by the S-MF method in the presence of various detergent types. The T4L-PS1NTF and PS1CTF proteins were co-purified by tag-affinity chromatography with the FLAG tag at the C-terminus of PS1CTF, in 50 mM Tris-HCl buffer (pH 7.0) containing 0.05% βDDM, 0.002% CHS, and 400 mM NaCl. (b) SDS-PAGE images of the T4L-PS1NTF•PS1CTF and T4L-PS1NTF•PS1CTF•Pen-2 complexes, produced by the S-MF method in the presence of Brij-78 or digitonin, and purified with the FLAG-tagged PS1CTF in the same manner as in (a). (c) The enzymatic activity of the T4L-PS1NTF•PS1CTF•Pen-2 complex of (b) with the intramolecularly quenched fluorogenic peptide probe. The fluorescence intensity increases per minute with and without the GXGD protease inhibitor, (Z-Leu-Leu)2 ketone, are shown with black and white bars, respectively. $C_{10}E_5$, pentaethylene glycol decyl ether; $C_{10}E_8$, octaethylene glycol decyl ether; $C_{12}E_6$, hexaethylene glycol dodecyl ether; OG, *n*-octyl-β-*D*-glucopyranoside; NG, *n*-nonyl-β-*D*-glucopyranoside; DM, *n*-decyl-β-*D*-maltopyranoside; UDM, *n*-undecyl-β-*D*-maltopyranoside; DDM, *n*-dodecyl-β-*D*-maltopyranoside; TDM, *n*-tridecyl-β-*D*-maltopyranoside; OG-NG, octyl glucose neopentyl glycol; DM-NG, decyl maltose neopentyl glycol; CyGlu-4, 4-cyclohexyl-1-butyl-β-*D*-glucopyranoside; CyMal-6, 6-cyclohexyl-1-hexyl-β-*D*-maltopyranoside; DMHM, 2,6-dimethyl-4-heptyl-β-*D*-maltopyranoside; CHAPS, 3-[(3-cholamidopropyl)dimethylammonio]-1-propanesulfonate; BSA, bovine serum albumin. Each experiment was performed once. The gel images of (b) were processed by cropping.

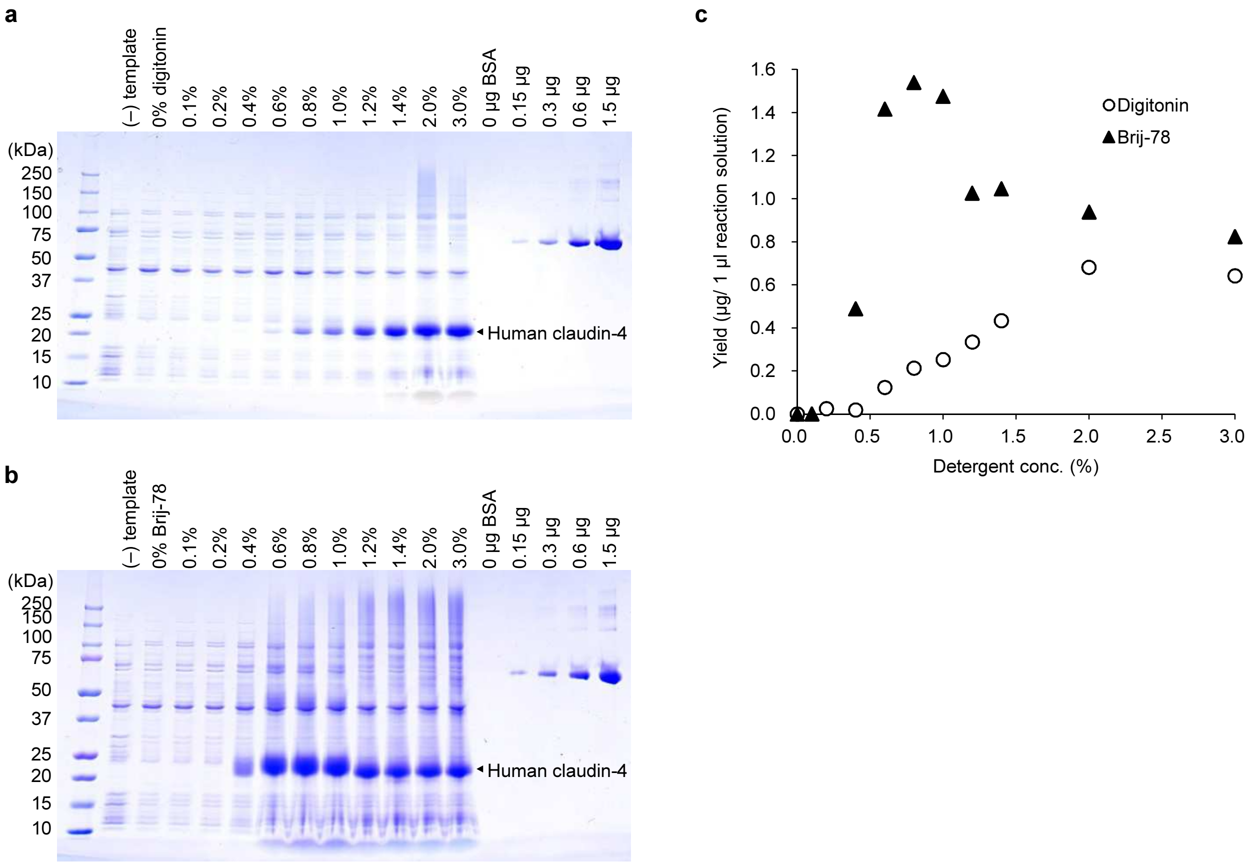

Supplementary Figure S3 | Production of human claudin-4 by the S-MF method. SDS-PAGE images of human claudin-4, produced by the S-MF method in the presence of various concentrations of digitonin (a) and Brij-78 (b), and purified by tag-affinity chromatography in 50 mM Tris-HCl buffer (pH 7.0), containing 0.05% βDDM, 0.002% CHS and 400 mM NaCl. Gels were stained with Coomassie Brilliant Blue. (c) Densitometry analysis of the bands of human claudin-4 in (a) and (b). The densities of the bands were measured with Image J (http://imagej.nih.gov/ij/), and the amounts of proteins were estimated from the calibration curve, using BSA as the standard. The amount of protein in each band is plotted against the detergent concentration. Each experiment was performed once.

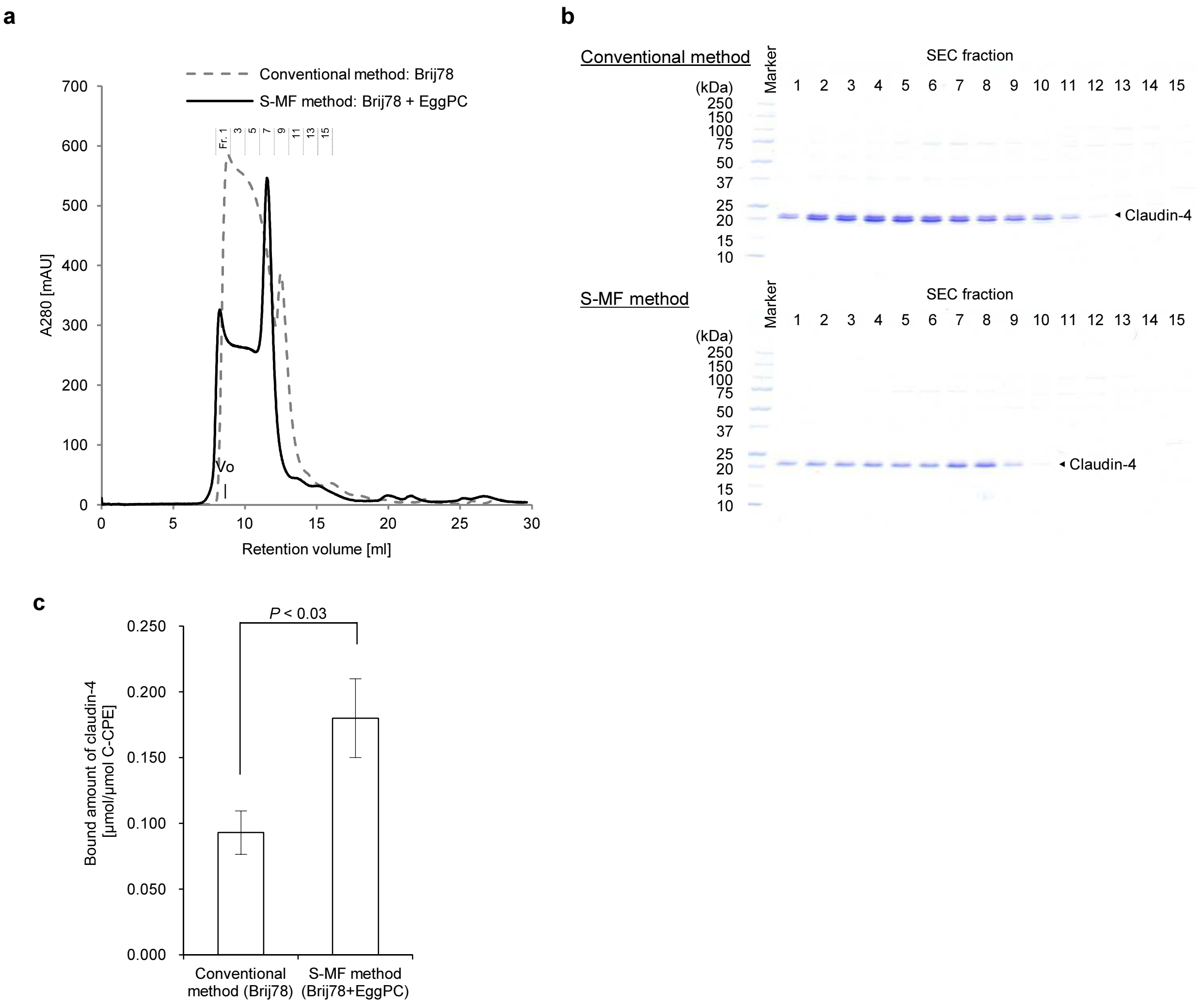

Supplementary Figure S4 | Comparison of the conventional method and the S-MF method for the production of human claudin-4. (a) SEC profiles after His-affinity chromatography of the cell-free produced human claudin-4 by the conventional method with 1% Brij-78 or by the S-MF method with 1% Brij-78 and 0.67% egg PC. The dashed and solid lines indicate the profiles of proteins produced by the conventional method and the S-MF method, respectively. (b) SDS-PAGE images of SEC fractions of (a). Gels were stained with Coomassie Brilliant Blue. (c) C-CPE binding analysis of the SEC peak fraction of human claudin-4 by the pull-down assay with GST-tagged C-CPE. The amount of bound claudin-4 was calculated from the densitometry analysis of the SDS-PAGE image, with means ± S.D. (error bars); *n* = 3.

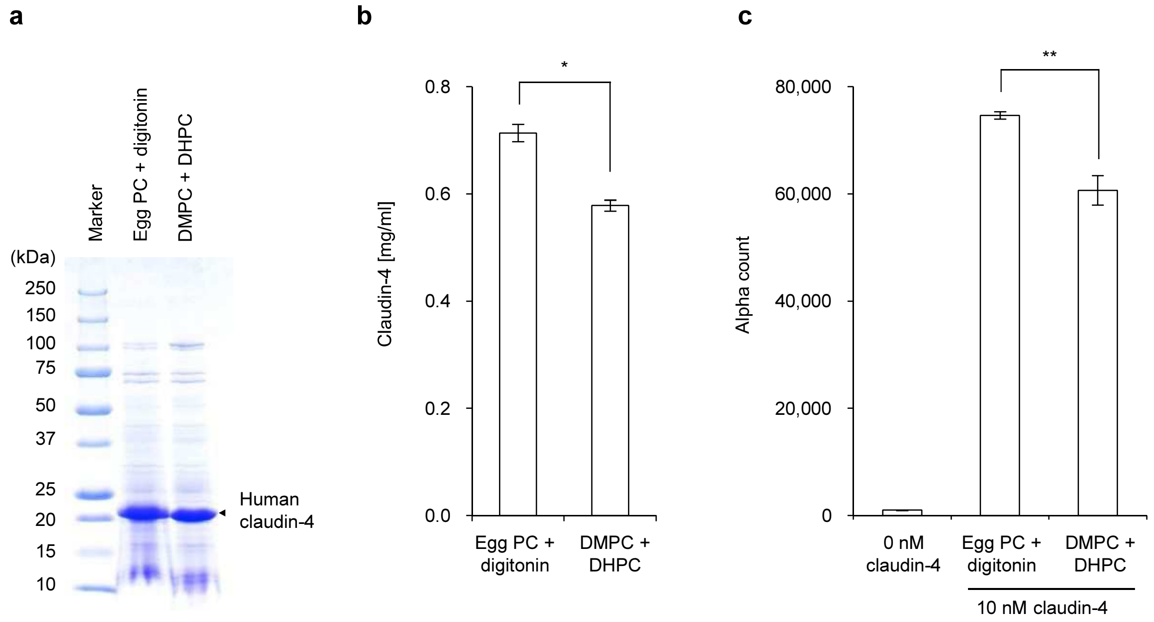

Supplementary Figure S5 | Comparison of the lipid/detergent conditions for the production of human claudin-4 by the S-MF method. (a) SDS-PAGE image of the IMAC-eluates of human claudin-4, produced by the S-MF method in the presence of 10 mg/ml digitonin and 6.7 mg/ml egg PC or 9.5 mg/ml 1,2-diheptanoyl-*sn*-glycero-3-phosphocholine (DHPC) and 6.7 mg/ml 1,2-dimyristoyl-*sn*-glycero-3-phosphocholine (DMPC). (b) The concentrations of human claudin-4 estimated from the densitometry analysis of (a), with means ± S.D. (error bars); $n = 3$. *, $p < 0.001$. (c) C-CPE binding analysis of the IMAC-eluates of human claudin-4 by AlphaScreen, using 10 nM claudin-4 and 40 nM GST-C-CPE, with means ± S.D. (error bars); $n = 4$. **, $p < 0.005$.

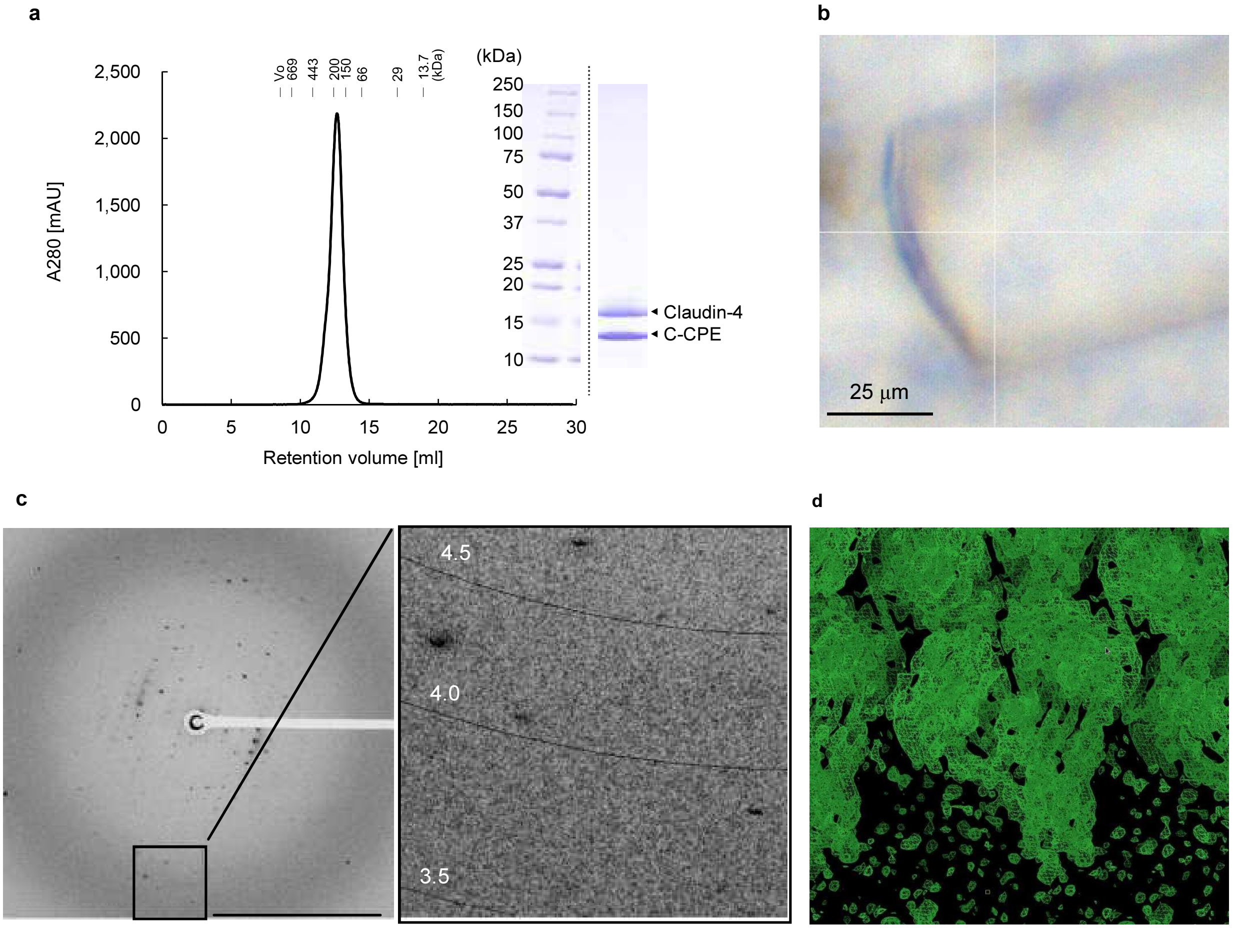

Supplementary Figure S6 | X-ray crystallography of the human claudin-4•C-CPE complex. (a) SEC elution profile of the human claudin-4•C-CPE complex. (b) The crystals of the claudin-4•C-CPE complex. (c) The diffraction images collected from the crystal of the claudin-4•C-CPE complex, using BL32XU of SPring-8. (d) The electron density map with the initial phase of the claudin-4•C-CPE complex at 4.5-Å resolution. The maps were drawn using the program COOT[52]. Gel images are representative of at least three experiments. Gel images in this figure were processed by cropping.
